# MicroRNA-10b promotes migration and invasion through KLF4 and HOXD10 in human bladder cancer

**DOI:** 10.3892/or.2022.8340

**Published:** 2022-05-31

**Authors:** Haibing Xiao, Heng Li, Gan Yu, Wei Xiao, Junhui Hu, Kun Tang, Jin Zeng, Wei He, Guohua Zeng, Zhangqun Ye, Hua Xu

Oncol Rep 31: 1832–1838, 2014; DOI: 10.3892/or.2014.3048

Following the publication of the above paper, an interested reader drew to the authors attention that, in Fig. 2 on p. 1835, which was designed to show how miR-10b promotes the migration and invasion of human bladder cancer cell lines *in vitro*, there appeared to be several overlapping panels such that certain of the data may have been derived from the same original sources, even though they were intended to show the results obtained under different experimental conditions. The authors have re-examined their original data, and have realized that the errors arose as a consequence of inadvertently misfiling and mishandling the data.

The corrected version of Fig. 2 is shown below. Note that these errors did not affect the overall conclusions reported in the study. All the authors agree to the publication of this corrigendum, and are grateful to the Editor of *Oncology Reports* for allowing them the opportunity to publish it; furthermore, they apologize for any inconvenience caused to the readership of the Journal.

## Figures and Tables

**Figure 2.**
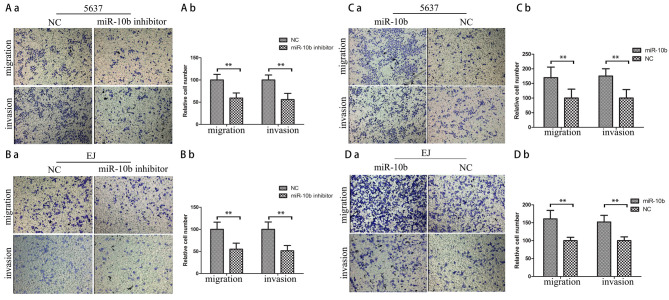
miR-10b promotes migration and invasion of human BC cell lines *in vitro*. (A and B) Transwell migration and invasion assays using 5637 and EJ cells transfected with miR-10b inhibitors or negative control (NC). Representative images are shown on the left, and the quantification of 10 randomly selected fields is shown on the right (means ± SEM; n=3; **P<0.01). (C and D) Transwell migration and invasion assays using 5637 and EJ cells transfected with miR-10b mimics or NC. Representative images are shown on the left, and the quantification of 10 randomly selected fields is shown on the right (means ± SEM; n=3; **P<0.01). BC, bladder cancer.

